# Uveitis Profile in Children and Its Impact on Vision at Queen Rania Children's Hospital

**DOI:** 10.7759/cureus.59136

**Published:** 2024-04-27

**Authors:** Marwan M Otoum, Noor M Al Adwan, Hala K Haddad, Mohammad N Al Aqarbeh, Mohammad Shihan, Ahmed Khatatbeh, Raed Alzyoud

**Affiliations:** 1 Ophthalmology, Royal Medical Services of Jordan Armed Forces, Amman, JOR; 2 Ophthalmology, King Hussein Hospital, Amman, JOR; 3 Ophthalmology, King Hussein Medical Center/Royal Medical Services, Amman, JOR; 4 Ophthalmology, Queen Rania Children's Hospital, Amman, JOR; 5 Ophthalmology, King Hussein Medical Center, Amman, JOR; 6 Immunology, Allergy, and Rheumatology, Queen Rania Children's Hospital, Amman, JOR

**Keywords:** juvenile idiopathic arthritis (jia), idiopathic, visual outcome, complications, pediatric uveitis profile

## Abstract

Aim: The aim of this study was to explore the patterns of pediatric uveitis and the types of ocular complications of uveitis and to determine the possible risk factors associated with visual impairment.

Method: This was a cross-sectional study conducted at Queen Rania Children's Hospital between June 2020 and June 2023. All children diagnosed with uveitis were enrolled in the study. After collecting data from the patients and reviewing their medical records regarding age, gender, and past ocular and medical history, the patients were subjected to a detailed ophthalmic exam including best-corrected visual acuity (BCVA). Anterior segment exam using the slit lamp, intraocular pressure exam using Goldmann applanation tonometry, and posterior segment exam using 78 and 90 diopter Volk lenses were performed. Patients with other ocular diseases that affected visions not related to uveitis were excluded from the study.

Results: A total of 82 children, accounting for 130 eyes, were enrolled in this study, with ages ranging from 2 to 16 years (mean age 10.5±4.3 years). Among them, 27 were males, constituting 32.9% of the participants. Unilateral uveitis was observed in 34 eyes, representing 26.2% of cases. The mean age of uveitis onset was 6.9±1.9 years, and the mean disease duration was 4.8±0.4 years.

The majority of cases i.e. 90.8% (n = 74) were non-infectious, with 92.3% (n = 76) classified as non-granulomatous and 79.2% (n = 65) categorized as chronic. Anterior uveitis was the most prevalent site of inflammation in 70.8% of cases (n = 58), followed by panuveitis in 20.0% of cases (n = 16), intermediate uveitis in 6.2% of cases (n = 5), and posterior uveitis in 3.0% of cases (n = 2). The cause of uveitis could not be identified in 40.0% (n = 33) of cases. Juvenile idiopathic uveitis emerged as the most commonly known disorder associated with uveitis in 40.0% (n = 33) of cases. Complications were identified in 52.3% (n = 43) of cases, with posterior synechiae being the most prevalent; 26.9% (n = 22) demonstrated an improvement in BCVA, while 21.5% (n = 18) experienced a decline in BCVA relative to the initial assessment

Conclusion: Pediatric uveitis tends to manifest as anterior, chronic, bilateral, and non-granulomatous. Higher frequencies of severe visual impairment are linked to panuveitis, infectious and granulomatous uveitis, early-onset, long-duration cases, and male gender. The use of biologics has a positive effect, significantly improving or preserving visual acuity.

## Introduction

Uveitis, an inflammatory condition affecting the uveal tract of the eye, poses a significant risk to vision and is a serious concern among children. Pediatric uveitis accounts for 10% of uveitis cases, with an incidence rate of 4.3 per 100,000 and a prevalence of 27.9 per 100,000 [1,2]. Alarmingly, over half of uveitis cases lead to ocular complications, and nearly one-third of afflicted children experience severe visual impairment [3]. Common culprits responsible for this impairment include cataracts, glaucoma, and macular edema [4,5].

While idiopathic origins predominate, uveitis in children can be associated with systemic diseases, with juvenile idiopathic arthritis ranking among the most prevalent [6]. Infectious causes have also been identified as contributors to pediatric uveitis. Children with uveitis present a diverse spectrum of symptoms, which can range from complete asymptomatic cases to those marked by redness, photosensitivity, excessive tearing, blurred vision, floaters, and photopsia [7]. Early diagnosis and prompt intervention are pivotal, especially for asymptomatic cases, to safeguard visual acuity. Additionally, managing any underlying systemic disorders, when present, is paramount for long-term ocular health.

The comprehensive approach to managing pediatric uveitis demands collaboration among pediatric immunologists, rheumatologists, and ophthalmologists to ensure effective treatment. The introduction of biologic agents and immunosuppressive therapies has heralded a promising era in pediatric uveitis management, offering encouraging outcomes [8]. However, vigilant monitoring for potential side effects and strict adherence to therapeutic regimens are crucial [9].

The principal objective of this study is to evaluate the uveitis landscape among children at Queen Rania Children's Hospital in Amman, Jordan with a focus on understanding the spectrum of uveitis profiles, potential complications, and visual outcomes associated with the disease.

## Materials and methods

This cross-sectional study was carried out at Queen Rania Children's Hospital in Amman, Jordan, spanning from June 2020 to June 2023. The study included all children diagnosed with uveitis based on their medical records. Data collection was meticulous, involving patient interviews and thorough reviews of medical records. Various key aspects were focused on, including the age of uveitis onset, duration, causes, type, and treatments received by the patients, including both local and systemic treatments, as well as any surgeries related to uveitis management.

Additionally, information from previous ocular examinations at the time of diagnosis and during the follow-up period was systematically collected, covering parameters such as best-corrected visual acuity (BCVA), anterior and posterior segment evaluations, and intraocular pressure measurements. The patients' medical histories were also examined to identify any systemic diseases associated with uveitis, along with details of systemic treatments administered.

A comprehensive ophthalmic examination, including BCVA assessment, slit lamp examination for the anterior segment, intraocular pressure measurement using Goldmann applanation tonometry, and posterior segment examination using specialized lenses, was conducted. The outcome of the ocular examination regarding the site of inflammation and the presence of uveitis complications was reported.

Patients with other ocular conditions affecting vision unrelated to uveitis or its complications and patients with insufficient detailed examination records were excluded, as were those who did not adhere to treatment regimens or follow-up appointments.

For data analysis, descriptive statistics, the Chi-square test, and T-test were employed using IBM SPSS Statistics for Windows, Version 29 (Released 2023; IBM Corp., Armonk, New York, United States).

## Results

A total of 82 children, accounting for 130 eyes, were enrolled in this study, with ages ranging from 2 to 16 years (mean age 10.5±4.3 years). Among them 67.1% were females. Unilateral uveitis was observed in 26.2% (n = 34 eyes) of cases. The mean age of uveitis onset was 6.5±1.9 years, and the mean disease duration was 4.8±0.4 years.

The majority of cases i.e. 90.8% (n = 74) were non-infectious, with 92.3% (n = 76) classified as non-granulomatous and 79.2% (n = 65) categorized as chronic. Anterior uveitis was the most prevalent site of inflammation in 70.8% of cases (n = 58), followed by panuveitis in 20.0% of cases (n = 16), intermediate uveitis in 6.2% of cases (n = 5), and posterior uveitis in 3.0% of cases (n = 2). Notably, 60.0% (n = 49) of cases were entirely asymptomatic at the time of diagnosis. Among symptomatic patients, redness in 19.2% of cases (n = 6) and photosensitivity in 19.2% of cases (n = 6) were the predominant symptoms. Visual impairment was identified in 9.2% (n = 3) of cases, while other infrequent symptoms 11.6% ( n = 4 ), such as floaters, photopsia, eye discomfort or pain, and lacrimation, were also noted.

Concerning etiology, the cause of uveitis could not be identified in 40.0% of cases. Juvenile idiopathic uveitis emerged as the most commonly known disorder associated with uveitis. The detailed spectrum of clinical conditions linked to uveitis is outlined in Table 1.

**Table 1:** Clinical conditions associated with uveitis

Etiology of uveitis	Number	Percentage
Idiopathic	52	40.0%
Juvenile idiopathic arthritis	52	40.0%
Behcet’s disease	13	10.0%
Tubulointerstitial nephritis-uveitis	1	0.8%
Toxoplasmosis	2	1.5%
Vogt-Koyanagi-Harada syndrome	3	2.3%
Herpetic uveitis	1	0.8%
Cytomegalovirus retinitis	3	2.3%
Tuberculosis	1	0.8%
Inflammatory bowel disease	1	0.8%
Sarcoidosis	1	0.8%
Total Number of eyes	130	100%

Complications were identified in 52.3% of cases, with posterior synechiae being the most prevalent, followed by band keratopathy and cataract as the primary complications associated with uveitis. The details of ocular complications are succinctly presented in Table 2. Surgical intervention became necessary in 51 eyes, and the various types of surgeries performed are outlined in Table 3.

**Table 2:** Types of ocular complications in uveitis

Complication	Number of eyes	Percentage
Posterior synechiae	42	32.3%%
Cataract /Pseudophakia/aphakia	33	25.4%
Band keratopathy	28	21.5%
glaucoma	18	13.8%
Vitreous haze	15	11.5%
Retinal scar	12	9.2%
Cystoid macular edema	9	6.9%
Ocular hypotony	2	1.5%
Retinal detachment	2	1.5%
Phthisis	2	1.5%

**Table 3:** Best-corrected visual acuity in relation to the most common uveitis profile at the time of uveitis diagnosis

Visual Acuity	Normal vision 63.8% (n = 83)	( <6/9 to 6/18) 20.0% (n = 26)	( <6/18 to 6/60 ) 10.0% (n = 13)	<6/60 6.2% (n = 8)
Site of inflammation	Anterior 79.5% (n = 66)	Anterior 61.5% (n = 16)	Pan uveitis 53.8% (n = 7)	Panuveitis 75.0% (n = 6)
etiology	Non-infectious 100% (n = 83)	Non-infectious 100% (n = 26)	Infectious 53.8% (n = 7)	Infectious 62.5% (n = 5)
Type of inflammation	Non-granulomatous 100% (n = 83)	Non-granulomatous 100% (n = 26)	Non-granulomatous 76.9% (n = 10)	Granulomatous 88.5% (n = 7)
chronicity	Chronic 78.3% (n = 65)	Chronic 76.9% (n = 20)	Chronic 84.6% (n = 11)	Chronic 87.5% (n = 7)
Age of onset	7.2	6.2	4.1	3.5
Duration of the disease	4.2	4.3	7.2	9.3
Associated disease	Juvenile Idiopathic Arthritis /idiopathic	Juvenile Idiopathic Arthritis	Behcet's Disease	Vogt Koyanagi Harada
gender	Female 81.9% (n = 68)	Females 73.1% (n = 19)	Females 53.8% (n = 7)	Females 50.0% (n = 4)

While 63.8% of eyes exhibited a best-corrected visual acuity (BCVA) of 6/9 or better, 20.0%, 10.0%, and 6.2% experienced mild, moderate, and severe visual impairment, respectively. A comprehensive breakdown of the BCVA spectrum at the time of presentation, relative to the uveitis profile, is encapsulated in Table 4.

**Table 4:** Types of surgeries performed among children with uveitis

Type of surgery	Number	Percentages
Cataract extraction	22	16.9%
Glaucoma surgery	13	10.0%
Chelation of band keratopathy	10	7.7%
Pars plana vitrectomy	5	3.8%
Periorbital injection	5	3.8%
Intra vitreal injection	3	2.3%

Approximately 51.5% of eyes exhibited a sustained best-corrected visual acuity (BCVA) in comparison to the BCVA recorded at the time of presentation. Conversely, 26.9% demonstrated an improvement in BCVA, while 21.5% experienced a decline in BCVA relative to the initial assessment. A detailed overview of the visual outcomes of eyes affected by uveitis, in correlation with both uveitis and patient profiles, is provided in Table 5.

**Table 5:** Final best-corrected visual acuity when compared to best corrected visual acuity at presentation

Best-Corrected Visual Acuity	Stable Best-Corrected Visual Acuity 51.5% of eyes (n = 67)	Improvement in Best-Corrected Visual Acuity 26.9% (n = 35)	Worsening in Best-Corrected Visual Acuity 21.5% (n = 28)
Site of inflammation	Anterior 89.6% (n = 60)	Anterior 71.4% (n = 25)	Pan uveitis 82.1% (n = 23)
etiology	Non-infectious 100% (n = 67)	Non-infectious 97.1% (n = 34)	Non-Infectious 60.7% (n = 17 )
Type of inflammation	Non-granulomatous 100% (n = 67)	Non-granulomatous 97.1% (n = 34)	Non-granulomatous 67.9% (n = 19)
chronicity	Chronic 80.6% (n = 54)	Chronic 77.1% (n = 27)	Chronic 78.6% (n = 22)
Age of onset (yrs)	7.5	7.1	3.5
Duration of the disease	3.5	3.9	9.2
Associated disease	idiopathic 46.3% ( n = 31 )	Juvenile Idiopathic Arthritis 48.0% ( n = 12 )	Vogt Koyanagi Harada 34.9% ( n = 10 ), Cytomegalovirus retinitis 34.9% ( n = 10 )
gender	Female 83.6% ( n = 56 )	Females 82.9% ( n = 29 )	Females 46.4% ( n = 13 )
Systemic steroids	26.9% ( n = 18 )	31.4%( n = 11 )	21 (75.0%) ( n = 21 )
Topical steroids	100% ( n= 67 )	100% ( n = 35 )	25 (89.3%) ( n = 25 )
Anti-metabolites	95.5% ( n = 64 )	88.5% ( n = 31 )	89.3% ( n = 25 )
Biologics	55 (82.1%)	33 (88.6%)	35.7% ( n = 10 )

## Discussion

Pediatric uveitis presents a complex challenge, carrying the potential for significant threats to vision if not promptly addressed. Unlike adult uveitis, cases in children often unfold asymptomatically, adding a layer of complexity to diagnosis. In the pediatric population, the presence of symptoms may indicate an advanced disease state or the presence of serious complications [9].

Adding to the complexity is the inherent difficulty in conducting thorough slit-lamp examinations in pediatric patients. The level of cooperation from children is notably lower than that of adults, making the examination of the eye a more intricate process [10,11]. Furthermore, the compliance of pediatric patients with prescribed treatment regimens tends to be less consistent compared to their adult counterparts.

Effectively managing pediatric uveitis requires heightened vigilance, early detection, and a tailored approach to both examination procedures and treatment strategies. This approach should consider the unique characteristics and potential complications associated with this age group.

In this study, the mean age of the patients was 10.5±4.3 years. The mean age of patients at the onset of uveitis was 6.5±1.9 years, which is comparatively lower than reported rates in Lebanon and Tunisia [9,12]. However, it aligns with rates observed in France and USA [11,13], indicating potential geographical or ethnic in certain regions. The majority of cases were chronic 79.2% (n = 65), anterior 70.8% (n = 58), bilateral 73.8% (n = 61), non-infectious 90.8% (n = 75), and non-granulomatous 92.3% (n = 76). Additionally, 32.9% (n = 27) of participants were males, and these rates closely resembled those found in regional and global studies [14].

The frequency of juvenile idiopathic arthritis (JIA) among systemic diseases causing uveitis varies widely, ranging from 0.0% in Japan to 61.0% in Finland [15,16]. Nonetheless, most global studies identify JIA as the most common systemic disease associated with uveitis [16]. In this study, JIA accounted for approximately 40.0% (n = 33) of pediatric uveitis cases. In contrast, Behcet’s disease emerged as the most common systemic disorder among children with uveitis, with a rate of 10.0% (n = 8). Notably, about half of the pediatric uveitis cases in various studies and approximately one-third of cases in the UK lacked an identifiable cause [17,18]. In our study, 40.0% (n = 33) of cases remained idiopathic, a proportion similar to findings in China, Japan, and Korea [1,2,19].

Despite typically affecting individuals in their 3rd and 4th decades, Behcet’s disease was the most commonly known etiology of pediatric uveitis after JIA in our study. Similar results were reported in Lebanon, Korea, and Turkey [2,9,15]. Notably, akin to adults and in contrast to other etiologies of uveitis, Behcet’s disease-associated uveitis was more prevalent in males than females.

Globally, the prevalence of infectious uveitis varies widely, ranging from 3.5% in the USA to 58.0% in Colombia [11,20]. In regional contexts such as Turkey, the incidence hovers around 15.6% [17]. Toxoplasmosis emerged as the predominant etiology for infectious uveitis in many studies, while Tuberculosis took precedence in India [21]. In our study, 9.2% (n = 8) of cases were infectious, with cytomegalovirus (CMV) being the most common infectious agent. This deviation from other studies can be attributed to the specialized unit for bone marrow transplants for immunocompromised patients at the hospital where the study was conducted.

Complications were identified in 52.3% (43) of cases at the time of the study and in 40.0% (n = 33) of cases at the time of diagnosis. This notably high percentage is largely due to pediatric uveitis being a predominantly asymptomatic condition, with symptoms often arising from the complications rather than the uveitis itself. However, there was a minimal and statistically insignificant increase in complication rates between the time of diagnosis and the time of the study, primarily attributed to the extensive use of biologics and immunomodulatory therapy at the time of diagnosis. In a study by Rosenberg et al., complications increased from 34.0% at diagnosis to 86.3% after three years [22].

Given that a majority of cases involved the anterior part of the eye, most complications were located in the anterior segment. Posterior synechia, cataracts, band keratopathy, and glaucoma were the most prominent complications. Surgical intervention was undertaken in 39.2% of cases, with cataract and glaucoma surgeries being the most frequently performed. This trend is likely attributed to the widespread and frequent use of topical and systemic steroid therapy [23,24].

In terms of BCVA, higher rates of pan uveitis, infectious etiology, early disease onset, and prolonged disease duration were found in eyes with moderate and severe visual impairment (p-value <0.05). Granulomatous uveitis was linked with severe visual impairment (P-value <0.05), while Juvenile Idiopathic Arthritis (JIA) exhibited a high association with normal or mildly impaired visual acuity (<0.05). Chronic uveitis was uniformly observed across all eyes with varying visual acuity spectrums (P value >0.05). Although females were predominant in eyes with normal or abnormal vision, their presence was much lower among patients with moderate and severe visual impairment (P-value <0.05).

While the majority of eyes demonstrated stable or improved BCVA, 21.5% (n =28) experienced a decline relative to the initial assessment. Eyes with pan uveitis, infectious etiology (specifically CMV), granulomatous uveitis, early-onset uveitis, long-duration uveitis, Vogt-Korangi-Harada etiology, and the use of systemic steroids exhibited higher rates of BCVA deterioration (p-value<0.05). Conversely, the use of biologics was strongly associated with stable or improved BCVA (p-value <0.05), aligning with previous studies highlighting the positive impact of biologics on visual outcomes in pediatric uveitis [8,25]. Male gender was found at significantly higher rates among eyes with deteriorating visual acuity (p-value <0.05).

The study results align with global and regional trends, depicting pediatric uveitis as predominantly anterior, chronic, bilateral, non-infectious, and non-granulomatous, albeit with variable frequencies. Variations in rates are attributed to genetic, geographical, ethnic, and methodological factors. Notably, CMV emerged as the most common infectious agent responsible for infectious uveitis in this unique context.

One of the limitations of our study is its exclusive conduct in a tertiary referral hospital. This may potentially impact the uveitis profile in terms of its etiology, anatomy, pathology, chronicity, and the types of treatment administered to patients. Given that mild or moderate cases were likely managed in other healthcare facilities, the findings might not fully represent the broader spectrum of uveitis cases in the Jordanian population. Another limitation of this study is its retrospective nature, which means we might not have access to detailed ocular examinations covering all aspects relevant to our study scope. This limitation could lead to a smaller sample size after the exclusion of those patients. Therefore, we recommend the implementation of a multicenter comprehensive prospective study to more thoroughly investigate the uveitis profile across various healthcare settings in Jordan.

## Conclusions

Pediatric uveitis typically presents as anterior, chronic, bilateral, non-infectious, and non-granulomatous. Poor visual outcome and visual deterioration were associated with pan uveitis, infectious uveitis, granulomatous uveitis, early-onset uveitis, prolonged disease duration, and male gender. In contrast, the utilization of biologics demonstrated a significant positive impact, leading to notable improvement or preservation of visual acuity.
